# Thalassemia in Guangxi: current situation and future prospects

**DOI:** 10.3389/fpubh.2026.1842288

**Published:** 2026-05-21

**Authors:** Lifang Liang, Sheng He

**Affiliations:** Guangxi Key Laboratory of Reproductive Health and Birth Defect Prevention, Guangxi Clinical Research Center for Birth Defects, Maternal and Child Health Hospital of Guangxi Zhuang Autonomous Region, Nanning, China

**Keywords:** Guangxi, model, prevention and control, strategy, thalassemia

## Abstract

Guangxi, one of the regions with the highest thalassemia burden worldwide, has a carrier rate of 23.89% in the general population. Over the past 15 years, it has established a government-led, multi-sectoral prevention network based on the principles of “government leadership, multi-department coordination, healthcare sector accountability, integration of medical and preventive services, and public participation.” This system has created an integrated “prevention–diagnosis–intervention–management” continuum supported by genetic testing and stem cell transplantation. Therefore, from 2010 to 2024, the birth rate of severe thalassemia decreased by 96.46%, and remained below 0.3 cases per 10,000 newborns for six consecutive years. This marks Guangxi’s successful transformation from a high-incidence area to a national demonstration zone. The success of the Guangxi Model stems from a paradigm shift from reactive clinical treatment to proactive public health strategy, deep integration of public health with clinical practice, and continuous technological and systemic innovation. Guangxi’s experience demonstrates that even in resource-limited settings, a systematic, multi-tiered strategy can effectively control genetic disorders, offering valuable lessons and a context-dependent reference for high-burden regions worldwide.

## 1 Introduction

Thalassemia is a monogenic hemolytic disorder caused by globin gene defects, leading to imbalanced globin chain synthesis and making thalassemia one of the most prevalent inherited disorders worldwide (1). It is primarily classified into *α*- and *β*-thalassemia based on the affected globin chain (2). Clinical severity ranges from asymptomatic carriers to severe forms. Patients with *α*-thalassemia intermedia (HbH disease) develop anemia, fatigue, and hepatosplenomegaly after infancy (3–5). Severe α-thalassemia (Hb Bart’s hydrops fetalis) often results in fetal demise or neonatal death (5). *β*-thalassemia intermedia presents with moderate anemia after age two and typically does not require regular transfusions (6, 7). In contrast, β-thalassemia major manifests as severe anemia, hepatosplenomegaly, and growth retardation within 3–6 months after birth, necessitating lifelong transfusions and iron chelation to prevent fatal iron overload, thereby imposing substantial burdens on families and healthcare systems (8–10).

Guangxi, located in southern China, is a multi-ethnic region inhabited by 12 indigenous groups, including the Zhuang (approximately 31.4% of the total population), Han, and Yao. As of the end of 2024, Guangxi had a resident population of about 50.13 million and a regional GDP of approximately 2.86 trillion yuan. As China’s only province sharing both land and maritime borders with ASEAN countries, Guangxi serves as a strategic gateway connecting Chinese and ASEAN markets. Epidemiological data indicate that the thalassemia gene carrier rate in Guangxi is as high as 23.89%, with rates in some areas exceeding 40%—significantly higher than in other southern Chinese provinces. This high carrier rate constitutes a large reservoir of pathogenic genes. As an autosomal recessive disorder, thalassemia presents considerable prevention and control challenges. Factors such as limited public awareness, low willingness to participate in screening, and traditional beliefs regarding marriage and childbirth may further increase the risk of pathogenic gene homozygosity. Additionally, the regional technical network remains underdeveloped, leading to potential missed or false detections among high-risk couples. Even when abnormalities are identified, some individuals may miss the optimal intervention window due to lack of access to precise diagnosis and genetic counseling. These multifaceted factors collectively pose a serious challenge to the region’s public health system (11).

To address this challenge, under the guidance of the National Health Commission, Guangxi took the lead in implementing the *Guangxi Thalassemia Prevention and Control Plan* in 2010. This was followed by the launch of the *Guangxi “Zero Births” Plan for Severe Thalassemia* in 2018 and the *Guangxi Three-Year Action Plan for Thalassemia Prevention and Control (2019–2021)* in 2019. Through over a decade of systematic efforts, a comprehensive prevention and control model characterized by “government leadership, departmental collaboration, healthcare sector accountability, integration of clinical and preventive medicine, and societal participation” has been gradually established and refined. In 2024, the birth rate of severe thalassemia in Guangxi is 0.08 per 10,000, representing a 96.46% reduction compared with 2.26 per 10,000 in 2010. This marks the sixth consecutive year that the birth rate has been maintained below 0.3 per 10,000, successfully achieving the “zero birth” target. From 2010 to 2024, a total of 13,701 cases of severe thalassemia were averted. This accomplishment has not only resulted in substantial savings in societal healthcare costs but has also safeguarded citizens’ health and employment rights, thereby contributing to the stability and equity of the labor market. These efforts have gained widespread recognition both domestically and internationally (12). Furthermore, Guangxi has actively promoted the internationalization of its prevention and control experience by participating in platforms such as the Asia Thalassemia Control Network and the China-ASEAN International Training Center for Thalassemia Prevention and Control, extending its approach to ASEAN countries and enhancing regional public health cooperation.

## 2 Current status of thalassemia in Guangxi

### 2.1 Epidemiological profile

Guangxi is a high-prevalence region for thalassemia in China, with the predominant forms being *α*- and *β*-thalassemia. The distribution of genotypes exhibits distinct geographical and demographic characteristics. In α-thalassemia, deletional variants are the main forms, with the most common types including *--SEA*, *−α3.7*, and *-α4.2* (13). Non-deletional mutations, such as Hemoglobin Constant Spring (*Hb CS*) and Hemoglobin Quong Sze (*Hb QS*), constitute a minority (14). *β*-thalassemia is primarily caused by point mutations, notably *CD41-42*, *CD17*, *IVS-II-654*, and *−28* (13). High carrier rates of thalassemia genes are found in both Zhuang and Han populations. However, the *α*-thalassemia carrier rate is significantly higher in Zhuang and Yao ethnic groups compared to the Han population (13). The *HKαα* genotype has been observed in both Han and Zhuang individuals in southern Guangxi (15). These patterns reflect the association between genotype distribution and population genetic backgrounds. As an autosomal recessive disorder, the prevalence of thalassemia is associated with factors such as population migration and genetic drift (16). Given Guangxi’s multi-ethnic composition, diverse geographical distribution, varied genetic backgrounds, and different marriage and childbirth customs, future prevention and control efforts necessitate the development of strategies that are both culturally sensitive and regionally targeted. This approach is crucial for achieving precision in public health governance (13).

### 2.2 Achievements of the prevention and control system

#### 2.2.1 Three-tier prevention and control system

Guangxi has established and refined a scientific and systematic three-tier prevention and control system. To improve the quality of the birth population, the region has set up 101 integrated premarital and reproductive health service platforms (17, 18). These platforms consolidate one-stop services including free premarital medical examinations, marriage registration, and preconception counseling for thalassemia. This initiative has maintained a consistently high premarital screening rate of over 99%, ranking among the highest in the nation, thereby integrating individuals of reproductive age into early risk identification and intervention from the outset. Furthermore, the system comprises 88 county-level thalassemia primary screening laboratories, 14 municipal-level thalassemia prenatal diagnosis sub-centers, 3 autonomous region-level thalassemia prenatal diagnosis centers, and 33 prenatal diagnosis institutions, forming a comprehensive service network that spans the provincial, municipal, county, and township levels (19). To enhance the accurate collection of maternal and child health service information and improve the quality and efficiency of related work, Guangxi independently developed the “Guangxi Maternal and Child Health Service Information Management System.” The system enables closed-loop management of the entire process from premarital screening and genetic diagnosis to prenatal diagnosis and medical intervention, ensuring meticulous tracking of high-risk couples and seamless connection between all key steps (20).

#### 2.2.2 Genetic counseling network

Guangxi has constructed a systematic genetic counseling framework for thalassemia. By providing precise risk assessment and scientific guidance on marriage and reproduction, this system assists high-risk couples in making informed reproductive decisions, contributing significantly to the reduction in the birth rate of children with severe thalassemia (12). To achieve efficient coordination across different stages of prevention and control, enhance the standardization of diagnosis and treatment, and narrow the gap between urban and rural healthcare services, Guangxi pioneered the launch of the nation’s first smart cloud platform dedicated to thalassemia prevention and control—“Yougui Yun (Superior Guangxi Cloud).” This platform integrates technologies such as 5G, cloud computing, and artificial intelligence, consolidating clinical diagnosis and treatment data with genetic information resources. Its functionalities encompass prenatal diagnosis, genetic counseling, remote consultations, quality control, and technical training. Moreover, leveraging the national “Cloud-based Maternal and Child Health” platform, Guangxi extensively conducts remote training, real-time guidance, multi-party consultations, and online education. This model enables primary-level medical institutions to access expertise from regional-level specialists, thereby improving the efficiency of grassroots diagnosis and treatment.

#### 2.2.3 International medical cooperation

In response to the “Belt and Road” Initiative, Guangxi has deepened multi-tiered cross-border medical cooperation (21, 22). As a pioneer in regional collaboration, Guangxi established the Asian Network for Thalassemia Control in 2004. A joint training center with Thailand’s Mahidol University, founded in 2018, had trained over 1,000 professionals from 13 countries by 2025. The 2019 establishment of the Fangchenggang International Medical Innovation Cooperation Zone provided a base for dispatching 170 medical teams to 28 countries and operating the “1369 Life Express,” a cross-border emergency rescue mechanism on the China-Vietnam border. Further expanding cooperation, Guangxi signed health agreements with four Vietnamese border provinces in 2025, launching a joint thalassemia and cataract project, and inaugurated the China-ASEAN Healthcare Cooperation Center (Guangxi) as an integrated hub for critical care, training, and research. As a regional hematopoietic stem cell transplantation center, Guangxi offers transplantation and gene therapy for severe thalassemia patients internationally, marking milestones with its first foreign pediatric transplant in 2024 and the first successful cross-border treatment of a Vietnamese patient in 2025. These outcomes validate the feasibility and effectiveness of the Guangxi strategy.

### 2.3 Therapeutic advances

#### 2.3.1 Hematopoietic stem cell transplantation

Guangxi has achieved breakthrough progress in the field of hematopoietic stem cell transplantation (HSCT) for thalassemia. In 2016, the region successfully performed its first haploidentical HSCT for a patient with severe thalassemia. In 2021, two pediatric patients were successfully cured using autologous HSCT combined with *γ*-globin gene reactivation technology. Presently, Guangxi has established itself as a transplantation center of significant regional influence for thalassemia. Since 2019, Guangxi has been systematically advancing HSCT capacity building across the region. This initiative involved the construction of 74 new dedicated transplantation units across 13 medical institutions, bringing the total number of units to 88. This expansion has formed an extensive and rationally distributed transplantation service network, substantially increasing the treatment capacity for children with severe thalassemia. As of December 2024, a total of 1,838 cases of HSCT for thalassemia had been completed in Guangxi, with the scale and clinical outcomes reaching internationally advanced levels (23). This single-center study only included patients with *β*-thalassemia (excluding *α*-thalassemia cases). Transplant types comprised matched sibling donor transplantation, unrelated donor transplantation, and haploidentical transplantation. Notably, despite continuous advances in curative technologies such as HSCT and gene editing, the core achievement of Guangxi’s 15-year program remains rooted in prevention.

#### 2.3.2 Gene therapy

Guangxi has been applying lentiviral transduction and CRISPR/Cas9 technologies to advance gene replacement and gene editing therapies for thalassemia, fundamentally modifying the genetic code underlying the disease. To date, 49 cases of thalassemia gene therapy have been completed in the region. A landmark achievement includes the successful conduct of the world’s first clinical study employing base editing for thalassemia treatment. Nine patients have undergone this therapy, all of whom have achieved transfusion independence. In recent years, gene therapy has been successfully used to treat two patients with hemoglobinopathies in Southeast Asia (one from Laos and the other from Malaysia) and two patients with sickle cell anemia in Africa. Furthermore, these efforts have enabled China to achieve its first successful case of gene therapy for sickle cell anemia.

## 3 Challenges and limitations

### 3.1 Patient management pressure

Despite significant progress in thalassemia prevention in Guangxi, the existing cohort of patients with intermediate and severe thalassemia continues to face ongoing management challenges, requiring substantial medical resources. Currently, it is estimated that approximately 12,000 thalassemia patients reside in Guangxi, with annual treatment costs exceeding 200 million yuan, imposing a significant impact on the healthcare budget. For patients in rural areas, access to standardized treatment remains difficult due to inconvenient transportation, heavy financial burdens, and a scarcity of local medical resources, highlighting the pronounced issue of uneven distribution of healthcare resources across regions (24). The “aging” of the patient population presents new challenges. As patients transition into adulthood, the incidence of complications such as endocrine disorders, cardiac iron overload, and hepatic fibrosis increases (10, 25), raising the demand for multidisciplinary care. Surveys indicate that adherence to iron chelation therapy is suboptimal (approximately 40%) among adolescent and young adult patients (26), leading to preventable complications and reduced life expectancy (10, 25, 26).

### 3.2 Disparities in primary care services

Significant disparities exist between urban and rural areas in terms of healthcare access and treatment quality. There is a clear imbalance in the distribution of medical resources across regions (27, 28). Patients in remote areas often require long-distance transfers, with some round-trip distances exceeding 300 kilometers. This increases the burden on families, negatively impacts treatment effectiveness, and raises overall medical costs (29). Healthcare institutions in rural areas frequently lack the necessary equipment for optimal thalassemia management (30). This includes magnetic resonance imaging for iron assessment, continuous subcutaneous deferoxamine pumps, and specialized laboratory tests. The absence of such resources creates substantial barriers to achieving optimal care and hinders the delivery of standardized treatment. Furthermore, there is a disproportionate geographical distribution of specialist medical personnel. These professionals are predominantly concentrated in provincial and municipal centers, while rural areas face a shortage of hematologists, transfusion medicine specialists, and genetic counselors. This scarcity constrains the capacity of primary-level facilities to diagnose and manage complex cases (28, 30).

### 3.3 Challenges in screening and follow-up for mobile populations and primary healthcare

The premarital and preconception screening rates among the mobile population are significantly lower than those of the local permanent residents. Migrant workers, due to their high mobility, demanding work schedules, and generally lower health awareness, often miss crucial screening and prenatal diagnostic opportunities. Furthermore, hindered by interoperability issues among provincial health information systems and the lack of cross-regional coordination mechanisms, mobile populations crossing provincial borders face even greater obstacles in accessing thalassemia screening and follow-up services. On the other hand, limited awareness of the hereditary risks of thalassemia among some rural residents, coupled with insufficient professional training for primary healthcare providers, adversely affects the accuracy of screening and the quality of genetic counseling services. These factors constrain the effective implementation of the three-tier prevention system (11).

### 3.4 Imbalanced resource allocation

Significant disparities in medical infrastructure exist across different regions of Guangxi, rooted in uneven levels of economic development and healthcare investment. Some county-level institutions, constrained by a lack of advanced genetic testing equipment, maintain a screening accuracy rate of approximately 85%. This percentage is far below the 95% or higher level achieved by provincial agencies, reflecting a significant difference in service quality. Furthermore, the *“Notice of the General Office of the People’s Government of Guangxi Zhuang Autonomous Region on Issuing the ‘14th Five-Year Plan’ for Guangxi’s Healthcare Service System*” highlights that per capita healthcare funding in affluent cities is approximately 2–3 times that of less developed areas. This structural imbalance in financial investment is the fundamental cause for the extreme disparity in the distribution of specialist physicians. For instance, the ratio of hematologists to population is about 1:50,000 in cities compared to 1:300,000 in townships and counties. This uneven distribution consequently limits equitable access to the treatment of complex cases and the dissemination of advanced therapies.

### 3.5 Limitations of the evidence base

The official statistical data from Guangxi on which this study relies may have limitations in terms of underreporting or incomplete registration. Although Guangxi has established a unified “Guangxi Maternal and Child Health Service Information Management System,” issues such as non-standardized or untimely case entry persist in practice. Furthermore, data quality varies across different cities and prefectures within the region, with data integrity in less economically developed areas often being lower than that in central cities. In addition, as a policy analysis review, this study draws most of its prevention and control indicators from government reports, news articles, and cross-sectional studies, lacking direct evidence from randomized controlled trials or cohort studies. The evaluation of causal effects of the policies relies primarily on before-after comparisons, making it difficult to completely exclude confounding factors—an inherent limitation of this type of policy review. Moreover, the policies and data from Guangxi discussed herein originate from a single region, carrying an inherent risk of geographic bias; the consistency of policy implementation and data recording standards may also exhibit heterogeneity across different time periods. It should be noted that, although the Guangxi model has demonstrated success in resource-limited settings, its direct extrapolation to other regions faces multiple constraints, including marked differences in healthcare systems and policy implementation capacities across countries, the influence of cultural and social factors on the acceptance of premarital screening and prenatal diagnosis, and disparities in economic resources that constrain screening coverage. Therefore, when adapting the Guangxi experience, other regions must make context-specific adjustments based on their local healthcare infrastructure, cultural background, and economic conditions.

## 4 Future directions and strategies

In 2024, the screening rates for thalassemia in Guangxi reached 99.99% for both premarital screening and screening of pregnant women at antenatal registration. The rate of prenatal diagnosis for identical genotypes was 94.20%, and the rate of pregnancy termination for fetuses with severe thalassemia was 99.63%. Analysis revealed that the failure to achieve 100% for these indicators is attributable to multiple factors. At the technical level, there exists a false-negative rate in initial routine blood screening, and some pregnant women registered for antenatal care too late to undergo prenatal diagnosis within the optimal window. At the individual level, lack of knowledge, low educational attainment, cultural customs, and personal refusal of services constituted major barriers. At the policy level, screening and interventions are based on the principle of voluntary participation and cannot be enforced. Additionally, incomplete coverage of the migrant population has contributed to a small number of cases that escaped follow-up.

### 4.1 Precision prevention system

#### 4.1.1 Strengthening coverage of mobile population screening

Intensifying Targeted Health Education: Collaborate with community organizations, enterprises, and other stakeholders to conduct regular public awareness campaigns on thalassemia prevention and control. These initiatives should disseminate information on screening policies, genetic risks, and the importance of early intervention (31, 32). For high-prevalence areas, develop culturally adapted promotional materials and disseminate them through targeted channels such as community bulletin boards and enterprise social media groups (31). Train community workers and corporate health officers to serve as “Thalassemia Prevention and Control Promoters”, empowering them to communicate key screening information effectively (31, 33, 34).

Refine Screening Follow-up and Dynamic Management Mechanisms: Establish a thalassemia screening database for the mobile population utilizing the “Guangxi Maternal and Child Health Information Management System.” Implement “one-to-one” targeted follow-up for individuals who have not undergone screening or are identified as high-risk, ensuring the timely implementation of prenatal diagnosis and intervention (17, 35). Set up screening registration points within community health service centers to provide appointment scheduling and reminder services, thereby reducing the missed screening rate.

Expand the Accessibility of Screening Services: Extend free screening services to areas with high concentrations of the mobile population, such as industrial zones, border ports, and labor markets. Deploy mobile screening stations in remote regions to provide integrated services encompassing screening, diagnosis, and genetic counseling, thereby shortening the service radius and enhancing accessibility.

#### 4.1.2 Strengthening secondary prevention

Integrating preimplantation genetic testing (PGT) into the three-tier prevention system and leveraging the development of a regional rapid prenatal diagnosis network can significantly enhance the accessibility of prenatal intervention services and shorten the decision-making cycle for high-risk couples. By establishing standardized operational protocols for prenatal diagnosis and genetic counseling, service quality can be homogenized and reliability ensured across medical institutions at different levels. As a key advancement, non-invasive prenatal testing (NIPT), based on the analysis of cell-free fetal DNA in maternal blood, offers a safer alternative to traditional invasive procedures while maintaining high diagnostic accuracy. This further reduces pregnancy-related risks. Furthermore, the extension and coverage of prenatal diagnosis services to county-level hospitals effectively alleviate the healthcare burden on families in rural areas, contributing to a substantive improvement in regional service accessibility.

### 4.2 Standardization of primary-level capacity

To comprehensively enhance the primary-level service capacity for thalassemia prevention and treatment, the implementation of the “County-Level Capacity Enhancement Plan” will significantly improve the accessibility of genetic diagnostic services within counties. This will be achieved through equipment upgrades, intensified personnel training, and the establishment of a telemedicine support system, effectively bridging existing service gaps. By unifying screening protocols and laboratory operational standards across healthcare institutions at all levels, consistent quality and reliability of test results will be ensured. Establishing a telemedicine platform that closely connects county-level hospitals with provincial-level experts will facilitate knowledge sharing, case consultations, and quality improvement activities, thereby strengthening the standardized case management capabilities at the primary level. Furthermore, the implementation of a regular supervision and quality assessment mechanism, including external quality control and proficiency testing, will guarantee that service standards are maintained at a high level across different regions.

### 4.3 Clinical translation of gene editing technology

CRISPR-Cas9 gene editing offers a novel therapeutic avenue for curing monogenic disorders such as *β*-thalassemia (36–38). The technology has demonstrated proof-of-concept efficacy in clinical trials for transfusion-dependent patients, particularly for those in whom conventional transplantation has failed or for whom a suitable donor is unavailable, with several patients achieving transfusion independence (39–42). However, to date, fewer than one hundred patients have received this treatment worldwide, and its long-term safety and efficacy remain to be verified through follow-up. Furthermore, its application to non-transfusion-dependent forms, including intermediate thalassemia and *α*-thalassemia subtypes, requires further advancement (43). To enhance the accessibility of this cutting-edge therapy in resource-limited settings, a multidimensional strategy integrating technological, economic, systemic, and governance dimensions is essential. This includes developing streamlined editing protocols and universal cell products to reduce technical barriers, leveraging technology localization to control costs, establishing regional collaborative networks to decentralize screening and follow-up while centralizing core technical operations, and formulating context-appropriate clinical-ethical guidelines. Such a systematic approach is crucial for translating treatment from the laboratory to the clinic and ultimately extending its benefits to a broader patient population (43). It must be emphasized, however, that gene editing remains an experimental therapy. At the current stage, large-scale application of this technology in Guangxi, or even across China, is not yet feasible and remains in the exploratory research phase. As such, it is not comparable to the well-established primary prevention strategy encompassing premarital screening, prenatal diagnosis, and genetic counseling.

### 4.4 Diversified support mechanisms

Patients with severe thalassemia require lifelong dependence on blood transfusions and iron chelation therapy, which imposes a continuous financial burden. While basic medical insurance covers a portion of these expenses, the out-of-pocket costs can still represent a substantial strain on patient families. Therefore, it is essential to integrate multi-sector social resources to jointly establish a sustainable, diversified support system (26).

Incorporating the diagnosis and treatment of thalassemia into the medical security system represents a crucial step in mitigating the financial risks faced by patient families. Building on this foundation, supplementary financing channels can be further expanded, including the establishment of special disease relief funds, encouragement of charitable donations, and enhancement of hospital-based financial assistance programs. The measures would provide additional support to families confronting catastrophic medical expenditures (44). Beyond economic aid, a systematic psychosocial support service should also be established, encompassing patient support groups, professional psychological counseling, and community rehabilitation programs (45–47). Such services aim to comprehensively address the mental well-being and social needs of patients and their families. Concurrently, workplace accommodation policies and improved educational support programs can better facilitate the social integration and participation of individuals with thalassemia in economic activities, thereby alleviating the long-term impact of the disease on both personal and societal development (48). Currently, adult patients with thalassemia rely on lifelong regular blood transfusion and iron chelation therapy, which incur substantial costs (49, 50), imposing a heavy financial burden on low- and middle-income families (51, 52). We therefore recommend further enhancing the coverage of blood transfusion and iron chelation therapy by increasing the reimbursement rate for thalassemia under the outpatient special chronic disease scheme to no less than 70% within the policy framework, and uniformly raising the annual maximum payment limit for eligible beneficiaries to 100,000 RMB. This would significantly improve treatment adherence and quality of life (53).

### 4.5 International cooperation framework

Leveraging the Belt and Road healthcare cooperation network, the China-ASEAN Forum on Thalassemia Prevention and Control is regularly convened. This platform promotes mutual recognition of prevention standards and coordination of control actions between the participating parties. Joint research projects addressing key regional challenges in thalassemia prevention and control are being advanced. These projects cover areas such as epidemiological surveys, cost-effectiveness analyses, and implementation research, generating evidence to inform relevant policies and practices. Furthermore, by establishing training programs and professional development opportunities for healthcare personnel from neighboring countries, regional capacities in thalassemia diagnosis, treatment, and management are being systematically enhanced. The development of standardized protocols for diagnosis, classification, and treatment not only ensures the homogenization of healthcare services across different regions but also establishes a foundation for conducting multi-site comparative effectiveness research.

## 5 International comparative analysis of thalassemia prevention models

**Figure 1:**
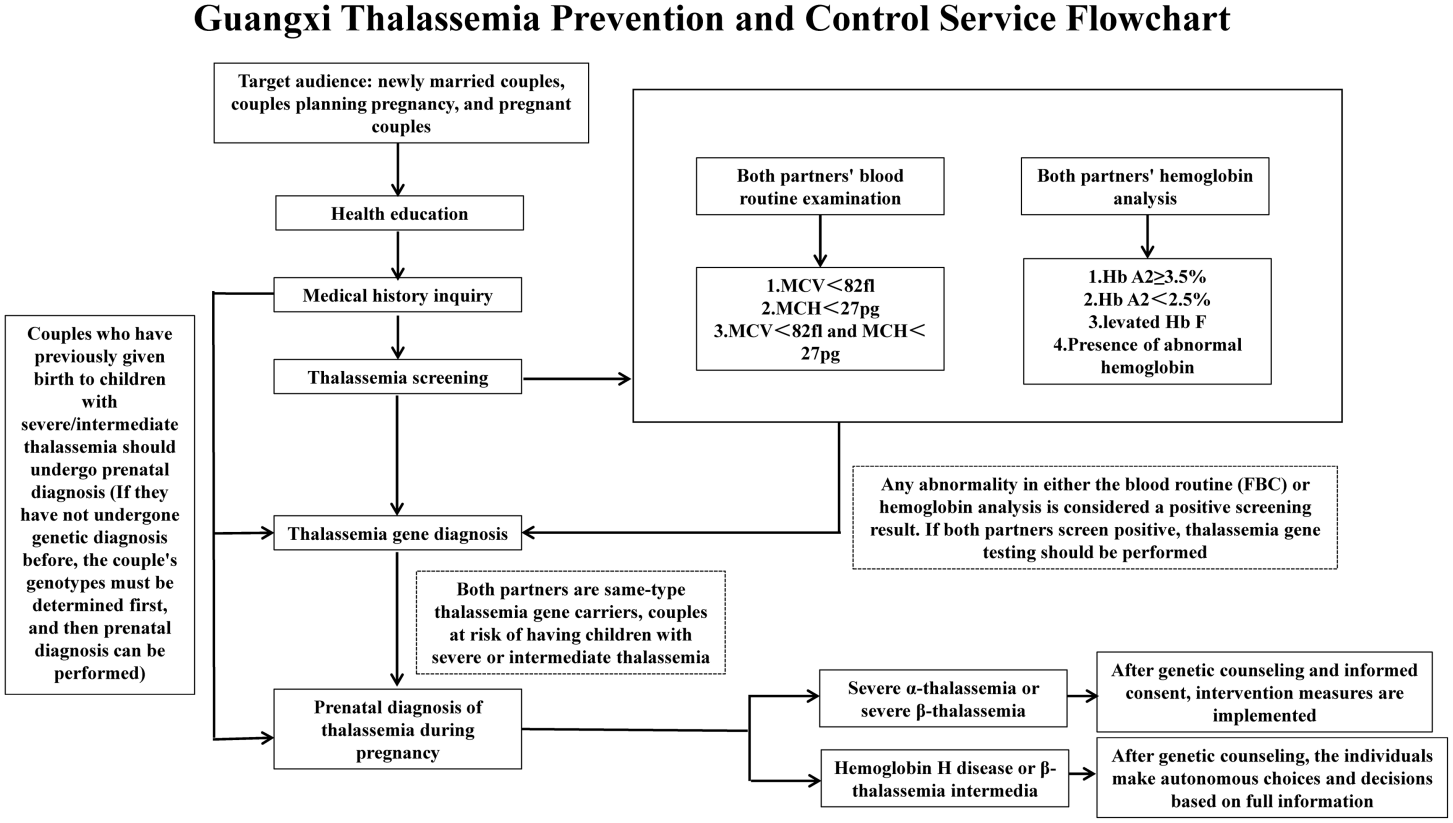
Guangxi thalassemia prevention control service flowchart.

In the global landscape of thalassemia prevention and control, Guangxi, Thailand, Cyprus, and Indonesia represent four distinct paradigms. The Guangxi model relies on strong administrative execution, constructing a closed-loop system of “four defense lines” that has achieved a premarital screening rate of >99% and reduced severe thalassemia births to <0.3 per 10,000, driven by government funding and administrative intervention (Figure 1). The Cyprus model is based on social consensus and religious collaboration, where decades of public education and Orthodox Church mobilization made voluntary premarital screening a social norm, achieving near-eradication without legal compulsion (54, 55). The Thailand model features regional collaboration and research integration, leading the Southeast Asian Thalassemia Alliance to unify screening standards, share data, and advance gene technologies through a “hub-and-spoke” network (56, 57). The Indonesia model exhibits a “nascent stage with external dependence” characterized by treatment insurance coverage (BPJS) with rural drug shortages, transplantation dependence on Thailand (with the first two pediatric transplants only completed in late 2023), and fragmented prevention efforts with rising neonatal cases (58, 59). Comparatively, Guangxi achieves “rapid and extensive” coverage through administrative enforcement; Cyprus achieves “deep and sustainable” results through cultural drivers; Thailand pursues “integrative and innovative” breakthroughs through regional leadership; and Indonesia is in transition “from treatment to prevention.” Together, these four paradigms demonstrate that successful thalassemia prevention and control require integrating government commitment, technological accessibility, and social mobilization, with their weights and pathways adaptively adjusted to local political systems, cultural traditions, and resource levels.

## 6 Conclusion

Guangxi’s endeavors in thalassemia prevention and control have established a successful paradigm, evolving from a regional practice to a model of universal relevance. This demonstrates that substantial outcomes are achievable through integrated control measures, even within resource-constrained settings. The region’s transformation from a high-prevalence area into a global innovation and demonstration center for thalassemia prevention charts a viable pathway with its proven strategies and concrete practices for other regions facing similar challenges worldwide. By integrating advanced medical technologies, a robust public health system, deep community engagement, and strong political commitment, Guangxi has created an exemplary framework adaptable to local conditions for controlling genetic disorders. From initial disease awareness campaigns to the establishment of a systematic screening network, and ultimately to achieving near-zero cases of severe neonatal illness, this process demonstrates that a coordinated, multi-layered, and sustained integrated strategy can steadily promote the achievement of public health goals. Looking ahead, Guangxi will continue to deepen its efforts in thalassemia prevention and control, aiming to progress from “zero severe neonatal cases” toward “zero cumulative cases.” This ambition reflects not only the region’s resolve but also contributes China’s insights and solutions to global public health, particularly in an era of increasing population mobility and interconnected health systems worldwide. Through continuous technological innovation, institutional optimization, and international cooperation, the Guangxi strategy is being refined, progressively enhancing its adaptability and relevance across diverse social contexts and health system conditions.
